# Smart garment for trunk posture monitoring: A preliminary study

**DOI:** 10.1186/1748-7161-3-7

**Published:** 2008-05-20

**Authors:** Wai Yin Wong, Man Sang Wong

**Affiliations:** 1 Department of Health Technology and Informatics, The Hong Kong Polytechnic University, Hung Hom, Kowloon, Hong Kong SAR, ProC

## Abstract

**Background:**

Poor postures of the spine have been considered in association with a number of spinal musculoskeletal disorders, including structural deformity of the spine and back pain. Improper posturing for the patients with spinal disorders may further deteriorate their pain and deformities. Therefore, posture training has been proposed and its rationale is to use the patient's own back muscles to keep the spine within the natural curvature. A posture training device may help to facilitate this therapeutic approach by providing continuous posture monitoring and feedback signals to the patient when "poor" posture is detected. In addition, the users of the device may learn good postural habits that could carry over into their whole life.

**Methods:**

A smart garment with integrated accelerometers and gyroscopes, which can detect postural changes in terms of curvature variation of the spine in the sagittal and coronal planes, has been developed with intention to facilitate posture training. The smart garment was evaluated in laboratory tests and with 5 normal subjects during their daily activities.

**Results:**

Laboratory tests verified that the accuracy of the system is < 1° and < 1.5° in static and dynamic tilting measurements respectively. The results showed that the smart garment could facilitate subjects to prevent prolonged poor postures of the spine, especially the posture of the lumbar spine in which at least 40% of the time in poor posture were reduced.

**Conclusion:**

The smart garment has been developed to be a portable and user-friendly trunk posture monitoring system and it could be used for collection of the trunk posture information and provision of instant feedback to the user if necessary for posture training purpose. The current pilot study demonstrated that the posture of normal subjects could be monitored and trained via this smart garment. With further clinical investigations, this system could be considered in some flexible spinal deformities such as scoliosis and kyphosis.

## Background

Poor postures of the spine, deviations from the "natural curvature of the spine", have been considered in association with a number of spinal musculoskeletal disorders, including structural deformity of the spine and back pain [1-5]. Spinal disorders could occur in different populations, including growing, working and aging populations. For instance, adolescent idiopathic scoliosis is the most common type and represents about 80% of idiopathic scoliosis. Apart from physical deformity of the spine, postural and proprioceptive dysfunctions are suggested to be the common defect of idiopathic scoliosis [6-9], which may deteriorate the spinal deformities. Low back pain always gives a major burden to the society as it associates with high costs of health care utilization, work absenteeism and disablement [10] and the lifetime prevalence range of low back pain is from 65% to 80% [11]. In working populations, occupational exposures such as heavy and/or repetitive lifting, fixed postures and prolonged seating, particularly in improper postures are related to low back pain [12]. Incidence of osteoporotic vertebral fractures is rapidly rising with aging in both sexes. Twenty-five percent of women who are with age > 50 years in the general population have one or more vertebral fractures resulting in loss of height and increased kyphosis (round back).

The multi-disciplinary rehabilitation concept of these spinal disorders include surgical, medical and orthotic interventions, and back muscle strengthening exercises to counteract postural deviations of the patients. For non-surgical and non-medical interventions, conventional orthotic interventions are to apply passive forces to the human body with orthosis for supporting the trunk alignments and controlling the deformities of the spine. Currently, rigid thoraco-lumbo-sacral orthoses (TLSO) are used for patients with progressive idiopathic scoliosis and soft lumbar corsets are used for patients with low back pain and elderly with osteoporotic vertebral fracture. However, the use of these external supports is limited by the factors such as poor appearance, bulkiness, physical constraint, cause of muscle atrophy that could lead to low acceptance and compliance ((([13-15]. Back muscle strengthening exercises are trying to strengthen the back muscle for keeping the trunk in upright posture with active muscular forces. However, the patients' compliance with the prescribed exercise interventions present a challenge, especially patients who are not self-motivated may not continue with the prescribed exercise programs [16,17].

Improper posturing for the patients with spinal disorders can further deteriorate their pain and deformities. Poor posture is defined as any prolonged deviations from the "neutral spine". Therefore, posture training has been proposed and its rationale is to use the patients' own back muscles to keep the spine within the natural curvature. Thus, the corresponding symptoms may be prevented with the awareness of their posture [2,18]. Postural training can facilitate the refinement of proprioceptive awareness of upright posture and prevention of deterioration of some spinal deformities such as scoliosis and osteoporotic vertebral fracture. A "reminder" for a good posture may be an acceptable prophylaxis for those at risk. A posture training device can help to facilitate this therapeutic approach by providing continuous posture monitoring and feedback signals to patients when "poor" posture is detected. In addition, the users of the device may learn good postural habits that could carry over into their whole life.

Based on this therapeutic approach, a posture training system has been developed by Dworkin et al. to handle patients with adolescent idiopathic scoliosis or hyper-kyphosis [2]. The system was used to monitor the spinal posture in terms of instantaneous spinal length continuously in real-time and to provide feedback signals to patients in order to correct their posture. However, there are some drawbacks of the device. It tracks the longitudinal and horizontal torso circumferences only and cannot provide any information directly related to spinal curvatures. The tension of the torso loop can cause discomfort and pressure abrasions at the gluteal cleft and groin area. Therefore, a more user-friendly and innovative portable posture training system, which can measure the parameters related to spinal curvature, should be developed for providing postural information of daily activity and improving the feasibility and effectiveness of posture training.

In the recent decade, many positional sensors have been developed for robotic, industrial and aerospace and biomedical applications. These sensors become smaller in size and better performance by using advanced circuit technology. Accelerometers and gyroscopes are commonly used to provide information on position and orientation in aerospace and robotic industries. For trunk motions analysis, these sensors also can be used to measure the kinematic parameters of body segments [19-27], including inclination relative to the gravity, linear acceleration and angular velocity. Therefore, a portable posture monitoring system can be built with these miniature sensors.

The aim of this study is to introduce a smart garment with integrated accelerometers and gyroscopes which can detect postural change in terms of curvature variation of the spine in the sagittal and coronal planes, and demonstrate the feasibility of the smart garment in guiding normal subjects to keep away from poor posture of the spine during daily activities.

## Methods

### Equipments

Two equipments were used in this study, namely (a) a smart garment and (b) a three-dimensional video-base motion analysis system. The former was used in trunk monitoring and the latter was used as a reference system to collect the data in laboratory test for evaluation:

(a) A smart garment consisted of three sensor modules, a digital data acquisition and feedback system, battery pack and a garment. Each sensor module (size: 22 mm × 20 mm × 12 mm, weight: 6 g) consisted of a tri-axial accelerometer (KXM52-Tri-axis, Kionix) and 3 uni-axial gyroscopes (Epson) orthogonally aligned, and assembled in a quasi-rectangular box. These three sensor modules were integrated into the elastic tight-fitting garment and connected to the digital data acquisition and feedback system. The data acquisition and feedback system (Size: 21 mm × 50 mm × 84 mm, Weight: 44.5 g) consisted of microcontrollers, memory with associated circuit and buzzer, was packaged into a plastic box. The system operated with 4 AAA size rechargeable batteries (Ni-MH type, 1100 mAh, 1.2 V) which packaged in a battery holder (Size: 50 mm × 55 mm × 12 mm, Weight: 82 g) and requires recharging after 8 hours operation.

(b) A three-dimensional video-base motions analysis system with 6 cameras operated with infra-red light source (Vicon 370) was used to monitor the three-dimensional coordinates of the retro-reflective markers with 60 Hz sampling rate.

### Laboratory tests

**Figure 1:**
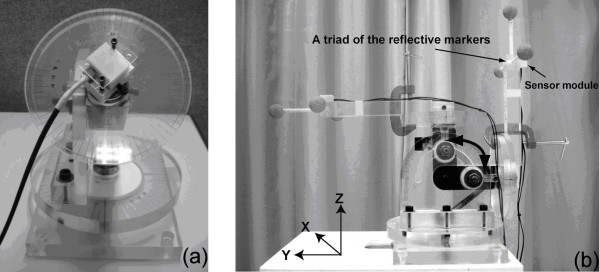
**Laboratory tests**. The experimental setting for (a) static tilting and (b) dynamic tilting measurements.

The accuracy of sensor modules for static tilting measurement was evaluated using a three-dimensional rotation alignment device. The alignment device can provide actual tilting angle with 1° increment. A sensor module was fixed on the device as shown in Figure 1a. The testing range was from ± 90° for the x- and y- axes. The sensor module was tilted along the x-axis with 5° interval in testing range and with pre-tilted angle along the y-axis, and vice versa. The pre-tilted angles were set as an interval of 20° in the range of ± 60° along the x- and y- axes. Three sets of measurement of each sensor module were collected. The error analysis of the measurement was tested in terms of root mean square (RMS) error and Pearson's correlation coefficient.

In dynamic tilting measurement, the accuracy of the sensor modules was evaluated using the motion analysis system along the x- and y- axes. The sensor modules connected to an interface board with power supply and adapters for connection of an analog-to-digital converter of the Vicon system for data collection. The experimental setup consisted of tilting platform and a triad with 3 reflective markers (Figure 1b). The orientation of reference coordinate system was derived from the three-dimensional coordinates of 3 reflective markers of a triad. The sensor module was affixed and aligned on the triad with the axis of the reference coordinate system. The tilting platform was tilted along the x- and y- axes manually in 5 tilting cycles with range of 90° for 5 trials.

### Trunk posture monitoring

**Figure 2:**
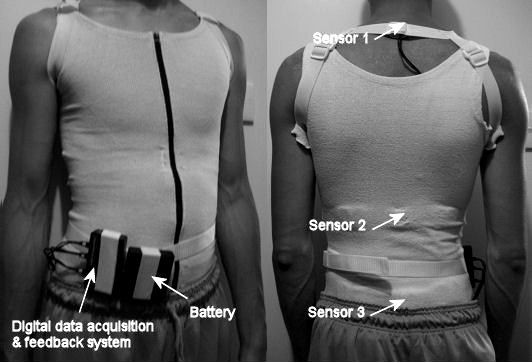
**A subject with the smart garment**. A subject with the smart garment.

**Figure 3:**
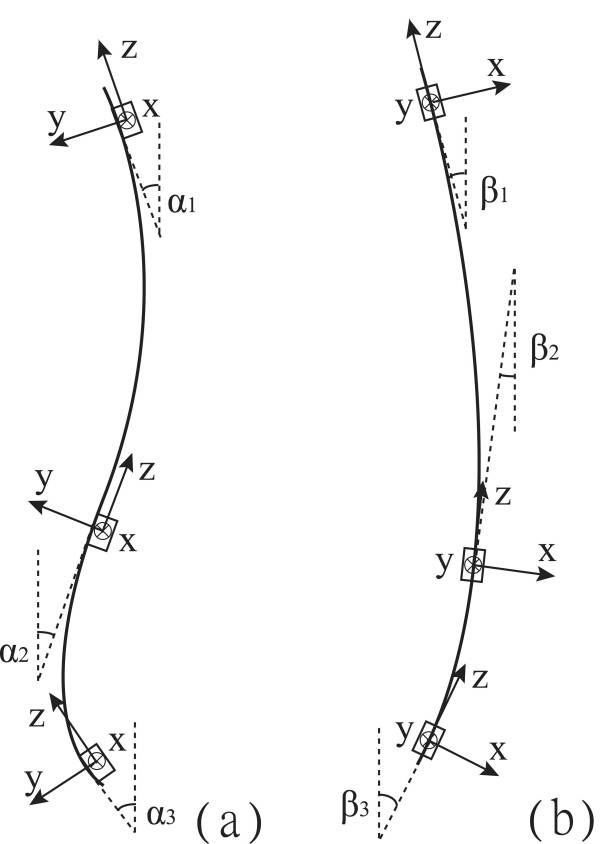
**The calculation of the trunk posture change**. The trunk postural changes were calculated with the data of the sensor modules and was defined as the change of inclination differences between angles α_(1,2,3) _and β_(1,2,3)_, respectively, which formed by the tangent and vertical line in the (a) sagittal and (b) coronal planes.

Five normal subjects (4 female & 1 male, age: 25.2 ± 4.8 years, weight: 50.5 ± 7.2 kg, height: 1.7 ± 0.09 m and BMI: 18.4 ± 1.1 kgm^-2^) used the smart garment in 4-day trials during daily activities with different protocols (Table 1). Written informed consents were obtained from all the five subjects prior to the experiments. Subjects wore the smart garment for 2 hours continuously a day during their leisure time at home (Figure 2). The garment should not be wearing during sleeping. The sensor modules were attached at the upper trunk (T1/T2), middle trunk (T12) and pelvis (S1). The trunk postural changes in the sagittal and coronal planes were estimated with output signals of the sensor modules in terms of curvature alteration measured between adjacent sensor modules and based on the concept of calculating the change of inclination angles difference between upper trunk and middle trunk for thoracic spine and between middle trunk and pelvis for lumbar spine in different trunk movements [28] (Figure 3). The measurement of the trunk posture using the sensor modules were evaluated and compared with the motion analysis system in previous study and reported in literature [29]. The sign of angle adopted as flexion and lateral bending to right were considered positive, and movement in opposite directions were represented by negative value for all data. It was calibrated in a neutral standing position for 1 minute before starting the posture monitoring. The neutral standing position was defined as standing against the wall and looking forward. In this position, the shoulder blades and the buttocks of the subject should be flat against the wall and the shoulders and the pelvis should be level, in order to perform the posture with the natural spinal curvature of the subject in standing positions.

**Table 1:** The protocol of trunk posture monitoring

		Target range		
Day	Feedback Status	Sagittal plane	Coronal plane	Tolerance time
Day 1	OFF	-	-	-
Day 2	ON	< 10°	± 10°	1 minute
Day 3	ON	< 5°	± 5°	1 minute
Day 4	OFF	-	-	-

The aim of the smart garment for posture training is to keep the tone off as long as the user can, thus the users will learn which postures will keep their back in natural spinal curvature. Eventually, the users can be trained to maintain the natural spinal curvature (good posture) more often as their posture habit. In this study, the mechanism was explained to the subjects in detail, but the exact interventional arrangement was not given to the subjects to prevent their perception bias. Feedback was provided in form of tone from a buzzer for 5 times (last for approximately 2 seconds) while the measured changes of trunk posture (trunk angles) at the thoracic and lumbar regions was out of the target range (for controlling flexion only in the sagittal plane, but both sides bending in the coronal plane) and last for longer than the specified tolerance time according to the protocol (Table 1). In this protocol, the feedback function of the smart garment was disabled on Day 1 and Day 4 for recording the posture information before and after 2-Days posture monitoring. The feedback function was enabled on Day 2 and Day 3 with two different thresholds for posture monitoring and demonstration of the effect of the feedback function. The tolerance time was set at 1 minute. The recording rate was set at 2 sets of measurements per minute. Tilting angles of the sensor modules and trunk angles of the thoracic and lumbar regions were record. The trunk angles between 4-day trials were tested using repeated measures ANOVA.

## Results

### Laboratory tests

The RMS error of the sensor modules is < 1° and Pearson's correlation coefficients is > 0.999 for the static measurement of couple-tilting angle in the range of ± 90° with pre-tilted angle in the range of ± 60° (Table 2). In the dynamic tilting measurement, the averaged RMS differences between the measurements of the sensor modules and motion analysis system are < 1.5° and the Pearson's correlation coefficients are > 0.999 (Table 3) for the measurements in the range of ± 90° and with RMS angular velocity < 40 deg/s along the x-axis (35.2 ± 1.9 deg./s) and y- axis (34.1 ± 1.7 deg./s).

**Table 2:** Averaged RMS difference in degree (mean ± standard deviation) in static tilting measurement

Pre-tilted angles	-60	-40	-20	0	20	40	60
X-axis	0.6 ± 0.2	0.6 ± 0.2	0.5 ± 0.1	0.4 ± 0.1	0.4 ± 0.1	0.5 ± 0.2	0.4 ± 0.2
Y-axis	0.4 ± 0.3	0.4 ± 0.2	0.4 ± 0.1	0.5 ± 0.1	0.6 ± 0.1	0.6 ± 0.2	0.5 ± 0.2

**Table 3:** Averaged RMS difference (degree) and Pearson's correlation coefficients (mean ± standard deviation) in dynamic tilting measurement

	RMS difference	Pearson's correlation coefficients
X-axis	1.3 ± 0.0	0.999 ± 0.001
Y-axis	1.5 ± 0.1	0.999 ± 0.001

### Trunk posture monitoring

**Figure 4:**
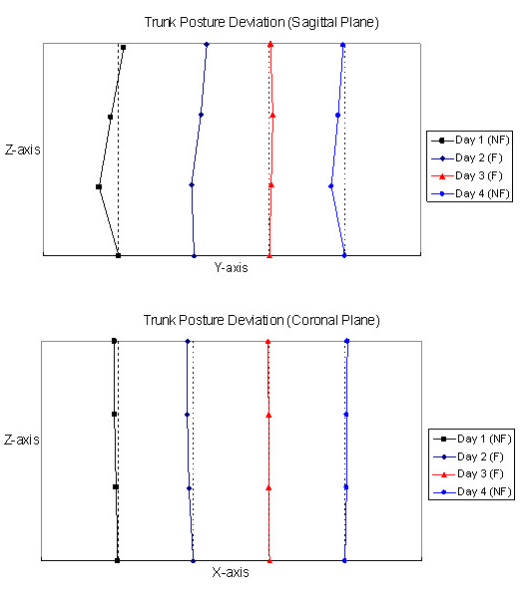
**The average trunk posture deviations in 4-day trunk posture monitoring**. The average trunk posture deviation from the neutral standing position of the subjects in 4-Day trial. Three line-segments represent the relative tilting of the 3 sensor modules to the neutral position. Dotted lines represent the posture in neutral position. The inter-segmental angles between 2 line-segments represent the trunk posture angles. (NF: without feedback, F: with feedback).

**Figure 5:**
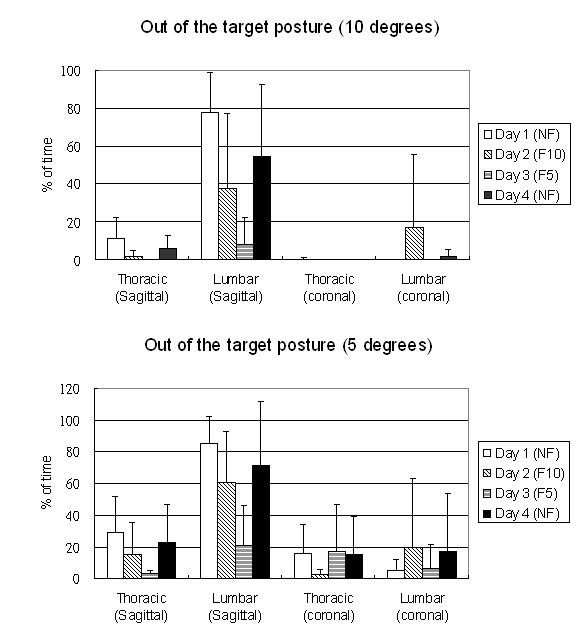
**The percentage of the time in out-of-target posture**. The percentage of the time in out-of-target posture. (NF: without feedback, F10: Feedback with 10 degrees threshold, F5: Feedback with 5 degrees threshold).

**Figure 6:**
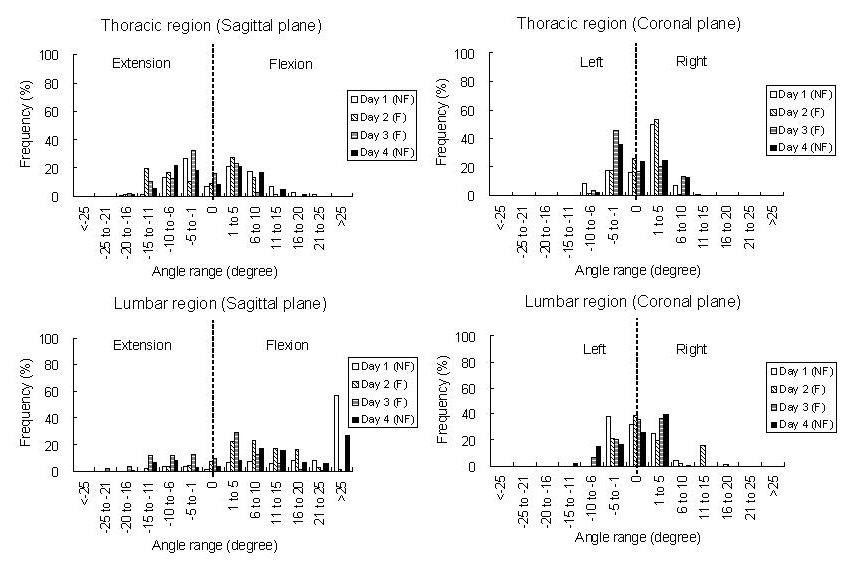
**The frequency distribution of the trunk postures**. The frequency distribution of the angles of the thoracic and lumbar curves in the sagittal and coronal planes. (NF: without feedback, F: with feedback).

For the 4-day trial, the averaged angles of the thoracic and lumbar curves in the sagittal and coronal planes are showed in Table 4. The trunk angle of the lumbar curve in the sagittal plane was significantly different among trials (p = 0.039) and found to be significantly smaller with the feedback function on Days 2 and 3 than those without the feedback function on Day 1 (p = 0.036 & 0.003 respectively). Figure 4 shows the trunk posture deviations of the subjects relative to the neutral position in the sagittal and coronal planes. In this figure, dotted lines represent the posture in neutral position and inter-segmental angles between 2 line-segments represent the trunk posture angles. Figure 5 shows the averaged percentage of the study time in which the angles of the thoracic and lumbar curves in the sagittal and coronal planes are in out-of-target posture. An average of 20% was found for the trunk angles in the coronal plane to be in out-of-target posture in the 4-day trial. For the sagittal plane, there was a trend in reducing the percentage of time of out-of-target postures, especially in the lumbar region with feedback function and reduced the target range. On Day 1, the lumbar curve was approximately 80% of the time in out-of-target posture, however, it was reduced to about 40% on Day 2 and 20% on Day 3 with feedback function. Figure 6 shows the frequency distribution of the angles of the thoracic and lumbar curves in the sagittal and coronal planes.

**Table 4:** The angles in the 4-day trunk posture monitoring.

	Sagittal Plane		Coronal Plane	
Trials	Thoracic curve	Lumbar curve	Thoracic curve	Lumbar curve
Day 1 (NF)	1.6° ± 1.9°	23.1° ± 11.3°	0.8° ± 3.2°	0.2° ± 1.0°
Day 2 (F)	-2.4° ± 3.3°	8.2° ± 6.6°	0.7° ± 1.6°	2.5° ± 5.8°
Day 3 (F)	-2.9° ± 1.5°	-0.7° ± 7.4°	0.1° ± 3.7°	-0.4° ± 2.4°
Day 4 (NF)	-0.4° ± 2.3°	14.5° ± 15.8°	0.4° ± 3.2°	-0.9° ± 4.1°
(p-value)	p = 0.443	p = 0.039	p = 0.927	p = 0.626

* The confident level of significance is 95% (p < 0.05) for repeated measures ANOVA

The angles (mean ± standard deviation) of the thoracic and lumbar curves in the sagittal and coronal planes. (Positive value represents flexion or right side bending; negative value represents extension or left side bending; NF: without feedback, F: with feedback)

## Discussion

For the 4-day trial of the trunk posture monitoring, all subjects reported that they were using computer and watching television and sitting more often than standing during monitoring period. The change of the trunk angles was more obvious in the sagittal plane than the coronal plane. The averaged value of the trunk angles in sagittal plane was smaller on the Days 2 and 3 than those on the Day 1. This might be due to the subjects straightening their spine more often with the feedback signals. It was found that the trunk angles were kept more frequently within the target range according to the protocol (Figures 5 &6). The results showed that the subjects tried to keep their trunk in extension more often on Days 2 and 3 (Figure 6) and they reported that they were sitting with the backrest of the office chair more often in these two days. The preliminary results demonstrated that the subjects could change their posture towards the target posture when feedback signals were provided. A reduction of approximately 26% of time which spent in "poor" posture of the thoracic spine is found in this current study (Figure 5) and it is comparable to those reported in the literatures [30,31]. For the lumbar spine, the reduction could be up to approximately 65% depended on the level of threshold (Figure 5). A decrease of the lumbar curve and the time of out-of-target posture occurred on Day 4 as compared with those on Day 1. This demonstrated that the subjects intended to keep the target posture after posture training with feedback function for two days. However, the effectiveness of the smart garment in posture training should be further verified in a long-term prospective clinical trial.

The choice of measurement parameter is essential in designing a posture monitoring system and should be interpreted easily. Posture training devices for treatment of scoliosis were developed by monitoring instantaneous torso length [2] and asymmetry of shoulder and pelvic levels [32,33] continuously. The measurement parameters of these devices are not directly related to the spinal curvature. In the current study, the choice of the measurement parameter was the change of the spinal profile curvature of the trunk. It is because the postural change of the trunk could be better interpreted in the spinal posture analysis and compared to the natural curvature of the spine via measuring curvature change instead of the other physical parameters.

Different numbers of sensors were used for monitoring and training people to improve trunk posture in the sagittal plane in literatures, included using one uni-accelerometer for monitoring the trunk tilting [34,35], two tri-axial accelerometers for monitoring postures of the thoracic spine [30] and six dual-axial accelerometers for monitoring alignments of the trunk [23]. Use of one accelerometer was able to detect the trunk tilting but not curvature change because of lacking reference signals from the distal portion of the trunk. Although more sensors could provide more information about the orientation of the spine, a good design should balance the accuracy and practicality, and not to cause considerable deviation from daily activities. In this current study, the results verified the feasibility of using three sensor modules to detect posture change of the thoracic and lumbar curves during trunk movements, in terms of spinal surface curvature change, in the sagittal and coronal planes simultaneously.

The rotational motion in the transverse plane could not be estimated using the smart garment in this current study because the accelerometer signals cannot provide the information about the motion in that plane to the auto-reset algorithm for estimation of the orientation of the sensor module. The possible solution of this problem is made use of the magnetic sensor to provide reference information, which sensing the magnetic flied of the earth, to auto-reset algorithm to correct the drifting problem of gyroscope signal [19,36]. The 3DOF orientation tracker (MT9, Xsens Technology B.V) has been developed with this technique for orientation measurement of human body segments, which can provide kinematic data, including 3D acceleration, 3D rate of turn and 3D earth-magnetic field. However, the measurement of the tracker will be affected at a magnetic environment which can full immunity to temporary magnetic disturbances for 30 seconds, the average static error was 1.4° and the dynamic error was 2.6° root means square in the magnetically disturbed experiments [36]. Further assessments in different magnetically disturbed surroundings including distance to ferromagnetic materials, types, mass and geometry, are needed for verifying this technique [36,37]. In future development of the system, it is a possible way to improve the orientation measurements with Kalman filter and combination of inertial sensors and magnetic sensors. At this moment, it may not be practical for using in daily situations. Although the additional sensor can provide more information of spine motion, the enlargement of the size of the sensor module after adding the extra sensor should be considered as the practical issue for monitoring during daily activities. In addition, the vertebral axial rotation could not be corrected even applying corrective forces to the scoliotic spine via the spinal orthosis [38-40]. It is difficult to be controlled via muscle forces during posture training. Therefore, the garment of this current study focuses on monitoring the posture change in the sagittal and coronal planes.

The inherent limitation of the surface methods is that the skeletal deformity of the spine cannot be assessed. Therefore, the smart garment was developed for monitoring the postural change of thoracic and lumbar regions relative to those in the neutral standing position as the general postural information as those in the literatures [2,33,35] rather than the deformities of the patients with posture deviations. The effectiveness of the system in posture training should be further evaluated with some suitable clinical cases of idiopathic scoliosis.

## Conclusion

The smart garment has been developed to be a portable and user-friendly trunk posture monitoring system that could be fixed onto the body for tracking trunk posture change in daily activities, and could be used for collecting the trunk posture information about daily postural habit of the users. The preliminary results demonstrated that the posture of normal subjects could be monitored and trained via this smart garment. The long term effect of this system is still under investigation.

## Competing interests

The authors declare that they have no completing interests.

## Authors' contributions

WY development of the smart garment, data acquisition, data analysis and report writing.

MS study design and coordination, data analysis and report writing.

All authors read and approved the final manuscript.
